# Zero-Heat Internal Thoracic Artery Harvesting Using Water Jet: An Experimental Study

**DOI:** 10.1016/j.atssr.2025.05.024

**Published:** 2025-06-30

**Authors:** Yoshinori Nakahara, Akira Marui, Kohei Sumi, Ryogen Yun, Makoto Ono, Tomohiro Iwakura

**Affiliations:** 1 Department of Cardiovascular Surgery, Sakakibara Heart Institute, Tokyo, Japan

## Abstract

**Purpose:**

To evaluate the feasibility and safety of internal thoracic artery (ITA) harvesting using water jet (WJ) technology.

**Description:**

The ERBEJET 2 (Erbe) hydrosurgical system was used to harvest ITAs in a skeletonized fashion. The device settings were optimized at 30 bar for selective tissue dissection while preserving vessels.

**Evaluation:**

Bilateral ITAs were harvested from 2 swine using WJ on one side and electrocautery on the other. Tissue samples (WJ n = 19, electrocautery n = 25) were histologically evaluated for coagulation (graded 0-4) and hemorrhage (graded 0-3). Coagulation was less frequent in the WJ group (26.3% vs 96.0%, *P* < .01) with lower grades (0.42 ± 0.77 vs 2.44 ± 0.82, *P* < .01). Hemorrhage occurred in all samples but was less severe in the WJ group (1.11 ± 0.32 vs 1.88 ± 0.97, *P* < .01). No thermal injuries were observed in either group.

**Conclusions:**

WJ harvesting of ITAs demonstrated less tissue damage compared with electrocautery, suggesting its potential as an alternative approach.

## Visual Abstract

**Figure:**
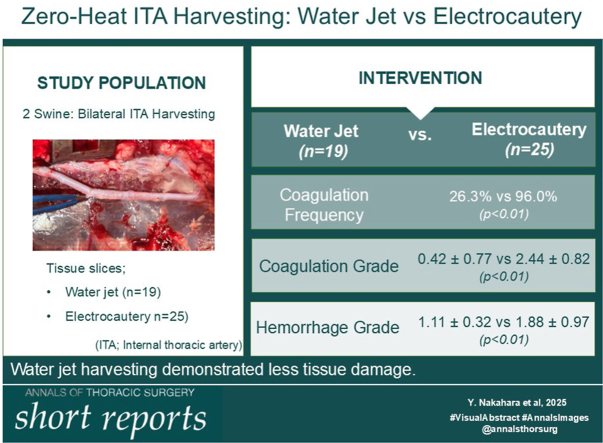

## Technology

**Figure 1:**
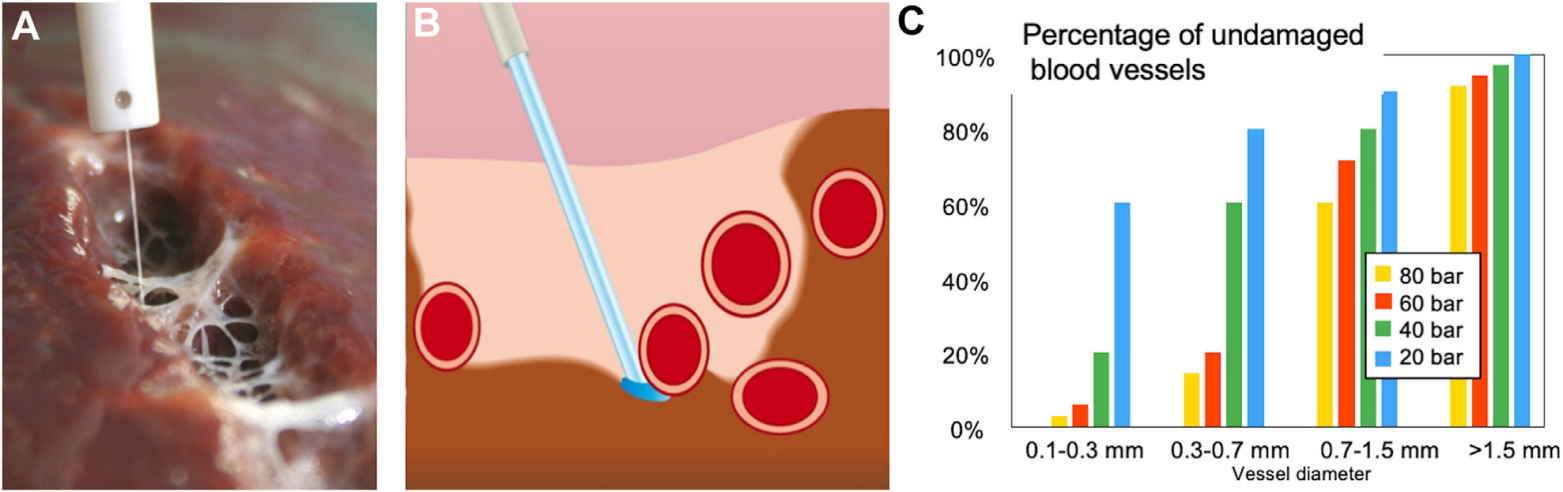
ERBEJET 2 (Erbe) hydrosurgical system. (A) Demonstration of liver dissection in a swine using ERBEJET 2, showcasing preserved small vessels. (B) Schematic diagram of the ERBEJET 2 device. (C) Relationship between jet pressure and vessel preservation in animal experiment. (Reproduced with permission, copyright Erbe Elektromedizin GmbH.)

In coronary artery bypass grafting (CABG), the left internal thoracic artery (ITA) to the left anterior descending artery bypass is the gold standard. Proper ITA harvesting is crucial for the success of the surgery, as dissection and thermal injury must be avoided. Currently, harmonic scalpels and electrocautery devices are used for ITA harvesting.^1^ However, these devices produce heat and require careful handling. The ERBEJET 2 (Erbe) is an advanced hydrosurgical system that uses high-pressure water jet (WJ) technology for cutting, dissecting, and removing tissues while preserving blood vessels (Figures 1A, 1B). A histologic study reported that WJ technology was the least traumatic technique.^2^ Furthermore, WJ allows for precise and gentle tissue dissection while preserving blood vessels and nerves,^3^ and elevates and separates tissue layers, creating fluid cushions that improve visibility and reduce bleeding during surgery (Figure 1C).^4^ In addition, WJ does not generate heat and is expected to avoid thermal damage to the tissue.

Therefore, the present study aimed to investigate the safety and efficacy of ITA harvesting using the WJ as compared with conventional electrocautery (EL) in terms of thermal injury.

## Technique

### Animal Model

The animal experiments were conducted in compliance with the Basic Guidelines for the Proper Conduct of Animal Experiments and Related Activities in Academic Research Institutions, under the guidance of the Ministry of Education, Culture, Sports, Science and Technology, adhering to humane principles. Two swine were used in the experiments. This study was approved by the ethics committee of Sakakibara Heart Institute on September 2, 2024 (approval number: 24-032).

### Surgical Procedure

In each experiment, a median sternotomy was performed under general anesthesia, and bilateral ITA harvesting was conducted. General anesthesia was induced with 3.0%-5.0% isoflurane inhalation, and sedation was maintained using midazolam and medetomidine. Sterile techniques were strictly followed throughout the procedure. Instruments were randomly assigned for ITA harvesting. In the first swine, the left ITA was harvested using WJ and the right ITA using electrocautery. In the subsequent swine, the sides were alternated, with the left ITA harvested using electrocautery and the right ITA using WJ. ITAs were harvested in a skeletonized fashion in both groups. The harvesting range extended from the level of the subclavian vein to the distal major bifurcation. These procedures were performed by 2 board-certified surgeons.

### Harvesting With Water Jet Device

The effect settings ranged from 10 bar to 80 bar, and 30 bar was chosen in this study. The WJ allows for the selective separation of tissues while preserving blood vessels. Although the artery and vein were successfully separated, further dissection using Metzenbaum scissors was needed due to the presence of tough fibrous tissue. Arterial branches were clipped for hemostasis. Supplemental Video 1 shows harvesting ITA with WJ.

### Harvesting With Electrocautery Device

The electrocautery device used was the VIO3 (ERBE) with effect settings at 3. Following the incision of the thoracic fascia, the ITA was dissected using the electrocautery. Arterial branches were coagulated and cut with the electrocautery, while larger branches were ligated with clips.

### Tissue Sampling and Analysis

The harvested ITAs were sectioned longitudinally by a pathologist, yielding a total of 44 slices. These specimens were processed with Elastica Masson staining, and a blinded pathologist evaluated them to assess coagulation and hemorrhage.

Coagulation was graded on a 5-point scale:

• Grade 0: No visible coagulation of the perivascular tissue
• Grade 1: Minimal coagulation, affecting <5% of the perivascular tissues
• Grade 2: Mild coagulation, affecting 5%-10% of the perivascular tissues
• Grade 3: Moderate coagulation, affecting 10%-20% of the perivascular tissues
• Grade 4: Severe coagulation, affecting >20% of the perivascular tissues

Hemorrhage of the perivascular tissue was evaluated on a 4-point scale:

• Grade 0: No visible hemorrhage
• Grade 1: Minimal hemorrhage, characterized by small isolated areas of extravasated erythrocytes
• Grade 2: Mild hemorrhage, presenting as multiple small areas or a few larger areas of extravasated erythrocytes
• Grade 3: Severe hemorrhage, exhibiting extensive areas of extravasated erythrocytes
Thermal injury, defined as tissue damage reaching the medial or intimal layers, was also assessed.

### Statistical Analysis

Statistical analysis was performed using R 4.4.0 software (R Project for Statistical Computing, R Foundation) and EZR (Saitama Medical Center, Jichi Medical University), which is a graphic user interface for R.^5^ Paired *t* tests and χ^2^ tests were used to compare the presence and grades of coagulation and hemorrhage.

## Clinical Experience

**Figure 2:**
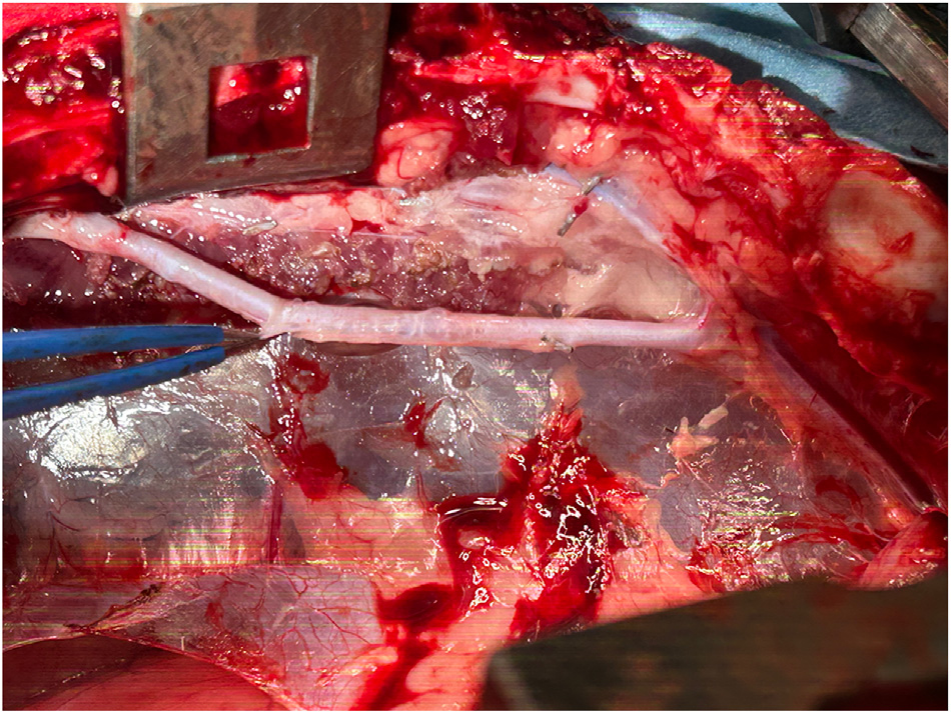
Intraoperative image of internal thoracic artery harvested using water jet. The internal thoracic artery graft is shown fully skeletonized with no visible evidence of coagulation or excess tissue.

**Figure 3:**
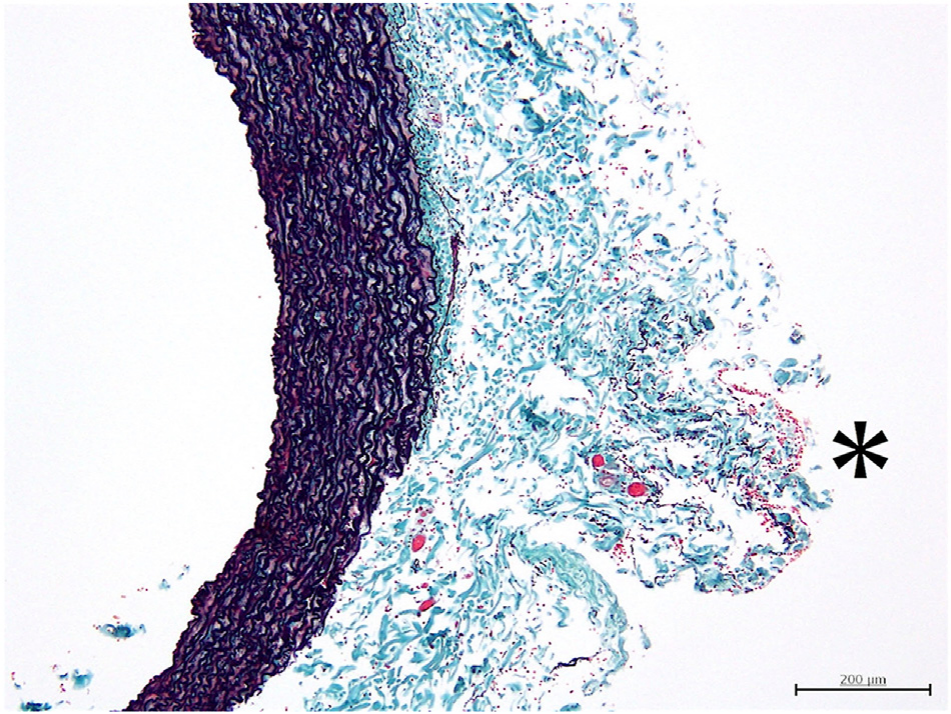
Histologic image of an internal thoracic artery harvested using water jet. The image shows minimal perivascular tissue damage, with little to no evidence of coagulation. Asterisk (∗) indicates areas of minor hemorrhage. (Elastica Masson staining, ×100 magnification.)

**Figure 4:**
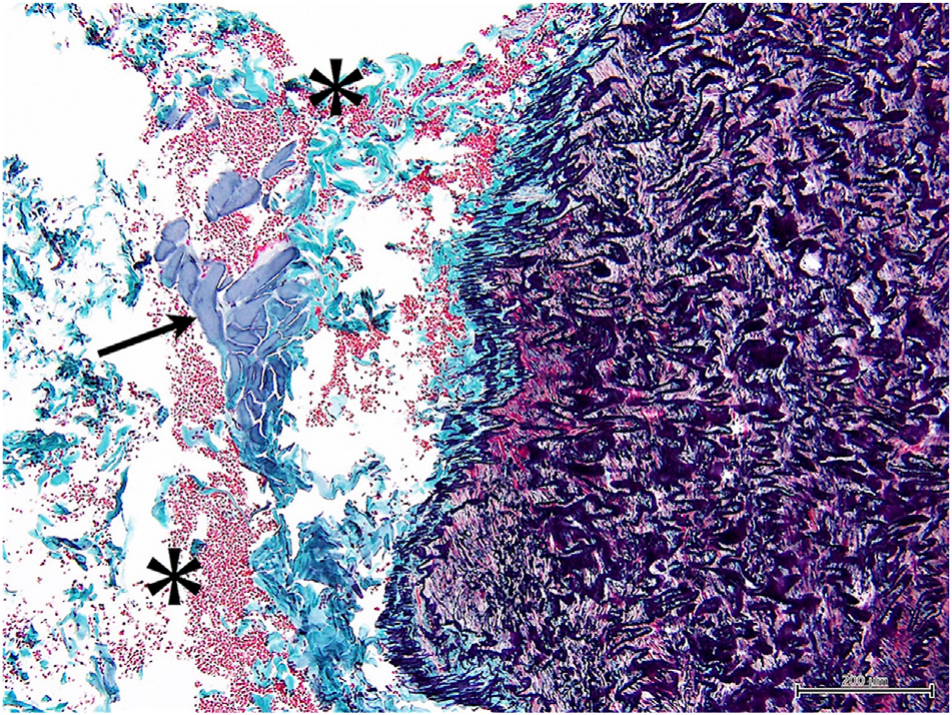
Histologic image of an internal thoracic artery harvested using electrocautery. The image demonstrates more extensive perivascular tissue damage, with visible signs of coagulation and hemorrhage. Asterisks (∗) indicate areas of hemorrhage, and black arrow points to regions of coagulation. (Elastica Masson staining, ×100 magnification.)

Bilateral ITA harvesting was successful in all experiments using both EL and WJ, with harvesting time in both groups being about 30 minutes (15 minutes and 28 minutes in the WJ group; 17 minutes and 20 minutes in the EL group). Analysis of 44 tissue slices (WJ group n = 19; EL group n = 25) revealed significant differences between the groups (Table 1). Coagulation was less frequent in the WJ group (5 of 19, 26.3%) compared with the EL group (24 of 25, 96.0%) (*P* < .01), with lower coagulation grades in the WJ group (0.42 ± 0.77 vs 2.44 ± 0.82, *P* < .01). Hemorrhage was present in all samples for both groups, but the severity was lower in the WJ group (grade: 1.11 ± 0.32 vs 1.88 ± 0.97, *P* < .01). Notably, no thermal injury was observed in either group. Intraoperative imaging (Figure 2) showed WJ-harvested ITAs were successfully skeletonized without evidence of coagulation or excess tissue. Histologic examination further confirmed these findings, with WJ-harvested ITAs showing minimal perivascular tissue damage (Figure 3), in contrast to the more extensive coagulation and hemorrhage observed in EL-harvested samples (Figure 4). As shown in Figures 3 and 4, no cellular disruption was observed in the intima and media layers of the internal thoracic artery, and neither penetrating damage nor cutting-induced injuries caused by the WJ or electric scalpel were detected.

**Table 1:** Comparison of Water Jet and Electrocautery Groups

Sample Grading	WJ Group (n = 19)	EL Group (n = 25)	*P* Value
**Coagulation**	5/19 (26.3)	24/25 (96.0)	<.001
Grade of coagulation	0.42 ± 0.77	2.44 ± 0.82	<.001
Grade 0	14 (73.7)	1 (4.0)	…
Grade 1	2 (10.5)	1 (4.0)	…
Grade 2	3 (15.8)	10 (40.0)	…
Grade 3	0	12 (48.0)	…
Grade 4	0	1 (4.0)	…
**Hemorrhage**	19/19 (100)	25/25 (100)	…
Grade of hemorrhage	1.11 ± 0.32	1.88 ± 0.97	.002
Grade 0	0	0	…
Grade 1	17 (89.5)	13 (53.0)	…
Grade 2	2 (10.5)	5 (20.0)	…
Grade 3	0	7 (28.0)	…
**Thermal Injury**	0/19 (0)	0/25 (0)	…

Values are presented as n (%) or mean ± SD.

EL, electrocautery; WJ, water jet.

## Comment

Our study aimed to explore the feasibility of using a WJ device for harvesting the ITA in a swine model. The results revealed 2 significant findings: The WJ enabled ITA harvesting without heat generation, and the procedure resulted in minimal hemorrhage and reduced coagulation compared with EL. These findings suggest that the WJ device may offer a safer alternative for ITA harvesting in CABG procedures.

The primary finding of this study is that the WJ enabled ITA harvesting entirely without heat generation, with the exception of the initial thoracic fascia incision. Consequently, coagulation in the perivascular tissue was notably reduced compared with electrocautery. This reduction can be attributed to the WJ's mechanism of action, which utilizes kinetic energy rather than thermal energy to dissect tissues. This approach avoids thermal injuries, thereby preserving the integrity of surrounding tissues. A previous study on the application of actuator-driven pulsed water jets for CABG demonstrated considerably lower thermal damage in harvested ITAs compared with conventional electrocautery.^4^ On the other hand, no thermal damage to the tunica media and tunica intima of the ITA was observed in either group in our study. This indicates that although EL is clinically safe, WJ offers an even safer alternative due to less superficial coagulation. The implications are significant: Minimized coagulation not only maintains the integrity of harvested grafts but also reduces the risk of complications such as tissue necrosis, inflammation, and particularly graft spasm.

Contrary to our initial expectations, the WJ device resulted in considerably less bleeding compared with electrocautery when harvesting the ITA. Although both groups experienced bleeding in all samples, the severity was markedly lower in the WJ group. This observation aligns with prior research indicating that WJ technology can effectively minimize tissue trauma and subsequent bleeding.^4^

The fine 120-μm tip of the WJ allows for precise movements, avoiding injury to vessels and the ITA itself during dissection. The WJ's settings selectively remove only tissues such as fat surrounding the vessels, thereby minimizing bleeding. An observational study reported successful preservation of the neurovascular bundle during anatomical nerve-sparing radical retropubic prostatectomy for patients with clinically localized prostate cancer, demonstrating the WJ's capability for precise dissection and better control of crossing vessels.^6^ This study was performed by surgeons with extensive ITA harvesting experience. Prior to these harvests, we conducted training with the WJ device using thoracic sections from swine cadavers, harvesting 10 ITAs to determine optimal output settings and dissection techniques. Despite this being our first live animal experience with the device, we completed the harvesting in acceptable timeframes. This suggests that the WJ device allows for precise movement and gentle dissection with a relatively short learning curve for surgeons already experienced in ITA harvesting.

The discontinuation of the Harmonic scalpel (Ethicon) in Japan as of April 2024 has created a need for alternative ITA harvesting methods in CABG. While the Harmonic scalpel had previously demonstrated superiority over EL by causing less thermal damage,^7^^,^^8^ this study suggests that WJ technology offers a safe, zero-heat alternative for ITA harvesting, potentially replacing the unavailable Harmonic scalpel. Additionally, this technique's benefits may extend to other delicate surgical procedures, potentially improving outcomes across various specialties.

This study has several limitations that should be addressed in future research:

1. The small sample size, with only 2 ITAs harvested with each device, limits the generalizability of our findings.
2. The ITAs in swine are thicker and more tightly surrounded by nearby structures than those of humans. This anatomical difference may affect the applicability of our results to human patients.
3. While this study focused on histologic evaluations, future research should assess the blood flow of the ITA grafts to comprehensively evaluate the technique.

Future studies should aim to address these limitations by increasing the sample size, comparing the WJ technique with other available harvesting methods, and including functional assessments of the harvested grafts. In particular, the relationship between WJ contact time, output settings, and ITA injury should be elucidated.

In conclusion, ITA harvesting using a WJ demonstrated the ability to harvest grafts without heat generation, resulting in significantly less coagulation and hemorrhage compared with EL. These findings suggest that the WJ might be a safer alternative for ITA harvesting in CABG procedures. Furthermore, a the reduced tissue damage observed with the WJ holds promise for potentially improved long-term graft patency, although further studies are needed to confirm this hypothesis.

## Freedom of Investigation

The water jet technology (ERBEJET 2) used in this study was borrowed from AMCO Inc. The authors declare that they had full control of the design of the study, methods used, outcome parameters, analysis of data, and production of the written report.

## Disclaimer

The Society of Thoracic Surgeons, The Southern Thoracic Surgical Association, and *The Annals of Thoracic Surgery Short Reports* neither endorse nor discourage the use of the new technology described in this article.

## References

[1] Masroor M., Zhou K., Chen C. (2021). All we need to know about internal thoracic artery harvesting and preparation for myocardial revascularization: a systematic review. J Cardiothorac Surg.

[2] Schurr M.O., Wehrmann M., Kunert W. (1994). Histologic effects of different technologies for dissection in endoscopic surgery: Nd:YAG laser, high frequency and water-jet. Endosc Surg Allied Technol.

[3] Sidorov D.V., Frank G.A., Mainovskaya O.A. (2014). Total mesorectal excision with water-jet dissection in patients with rectal cancer: surgical and morphological aspects. Colorectal Dis.

[4] Suzuki T., Kawamoto S., Nakagawa A. (2018). Application of actuator-driven pulsed water jet for coronary artery bypass grafting: assessment in a swine model. J Artif Organs.

[5] Kanda Y. (2013). Investigation of the freely available easy-to-use software 'EZR' for medical statistics. Bone Marrow Transplant.

[6] Fernández De la Maza S., Conrad S., Graefen M. (2002). Early clinical experience with water-jet dissection (hydro-jet) during nerve-sparing radical retropubic prostatectomy. Minim Invasive Ther Allied Technol.

[7] Higami T., Maruo A., Yamashita T. (2000). Histologic and physiologic evaluation of skeletonized internal thoracic artery harvesting with an ultrasonic scalpel. J Thorac Cardiovasc Surg.

[8] Kaneyuki D., Patil S., Jackson J. (2023). Ultrasonic scalpel versus electrocautery for internal mammary artery harvesting: a meta-analysis. Gen Thorac Cardiovasc Surg.

